# Effects of neutralizing antibodies on escape from CD8^+^ T-cell responses in HIV-1 infection

**DOI:** 10.1098/rstb.2014.0290

**Published:** 2015-08-19

**Authors:** Paul S. Wikramaratna, José Lourenço, Paul Klenerman, Oliver G. Pybus, Sunetra Gupta

**Affiliations:** 1 Department of Zoology, University of Oxford, Oxford OX1 3PS, UK; 2 Nuffield Department of Clinical Medicine, University of Oxford, Oxford OX3 7BN, UK

**Keywords:** HIV, AIDS, mathematical model, neutralizing antibodies, T-cell escape

## Abstract

Despite substantial advances in our knowledge of immune responses against HIV-1 and of its evolution within the host, it remains unclear why control of the virus eventually breaks down. Here, we present a new theoretical framework for the infection dynamics of HIV-1 that combines antibody and CD8^+^ T-cell responses, notably taking into account their different lifespans. Several apparent paradoxes in HIV pathogenesis and genetics of host susceptibility can be reconciled within this framework by assigning a crucial role to antibody responses in the control of viraemia. We argue that, although escape from or progressive loss of quality of CD8^+^ T-cell responses can accelerate disease progression, the underlying cause of the breakdown of virus control is the loss of antibody induction due to depletion of CD4^+^ T cells. Furthermore, strong antibody responses can prevent CD8^+^ T-cell escape from occurring for an extended period, even in the presence of highly efficacious CD8^+^ T-cell responses.

## 1. Introduction

Infection with HIV-1 typically commences with a large peak in viraemia and a significant depletion of the host's CD4^+^ T-cell population [1]. Several lines of evidence [2] suggest that CD8^+^ T-cell responses play an important role in the initial control of viraemia and the subsequent establishment of a stable ‘set-point’ viral load which may be maintained for many years, while CD4^+^ T-cell counts continue to fall. However, efforts to explain the eventual breakdown of virus control as a consequence of changes in CD8^+^ T-cell responses have met with little success. Strong, broadly directed and high-avidity γ-interferon positive CD8^+^ T-cell responses appear to persist in late-stage disease [3,4], and there is no correlation between CD4^+^ T-cell count and either the number of circulating anti-HIV CD8^+^ T-cells [5] or CD8^+^ T-cell-mediated lysis of infected cells [6]. Establishing consistent correlations between CD8^+^ T-cell function and viraemia has also proved difficult [7], and there is no apparent prognostic link between CD8^+^ T-cell functionality in early infection and AIDS survival time [8]. Yet, it is clear that HLA class I alleles have the effect of delaying progression to AIDS [9–13], suggesting that CD8^+^ T-cell responses continue to have a role in the maintenance of HIV-1 control beyond the early stage of infection.

By contrast with CD8^+^ T-cell responses, neutralizing antibody (NAb) responses do not typically reach detectable levels until several months after infection [14,15] and the high degree of variability of the viral envelope protein [16] is commonly used to question their utility in controlling infection (e.g. [17]). Yet, a number of early studies implicate the maintenance of a strong autologous antibody response in avoiding progression to AIDS [18–21], and depletion of B cells in humans [22] and non-human primates [23,24] has been shown to lead to increased viraemia and decline in autologous antibody responses. Furthermore, it has been demonstrated that NAbs can exert potent anti-viral effects at low or even undetectable titres in both humans [25] and in non-human primate models [26].

Here, we reconcile these conflicting observations using a model in which virus control is achieved by a combination of short-lived responses against CD8^+^ T-cell epitopes as well as long-lived antibodies to more diverse surface antigens. We use this framework to show antibody responses can also retard escape from CD8^+^ T-cell responses and lead to strong fluctuations in the frequency of CD8^+^ T-cell escape mutants during the course of infection. Escape from CD8^+^ T-cell responses accelerates disease progression; however, the ultimate breakdown of virus control is linked to the loss of antibody induction due to depletion of CD4^+^ T cells.

## 2. Model structure

**Figure 1.**
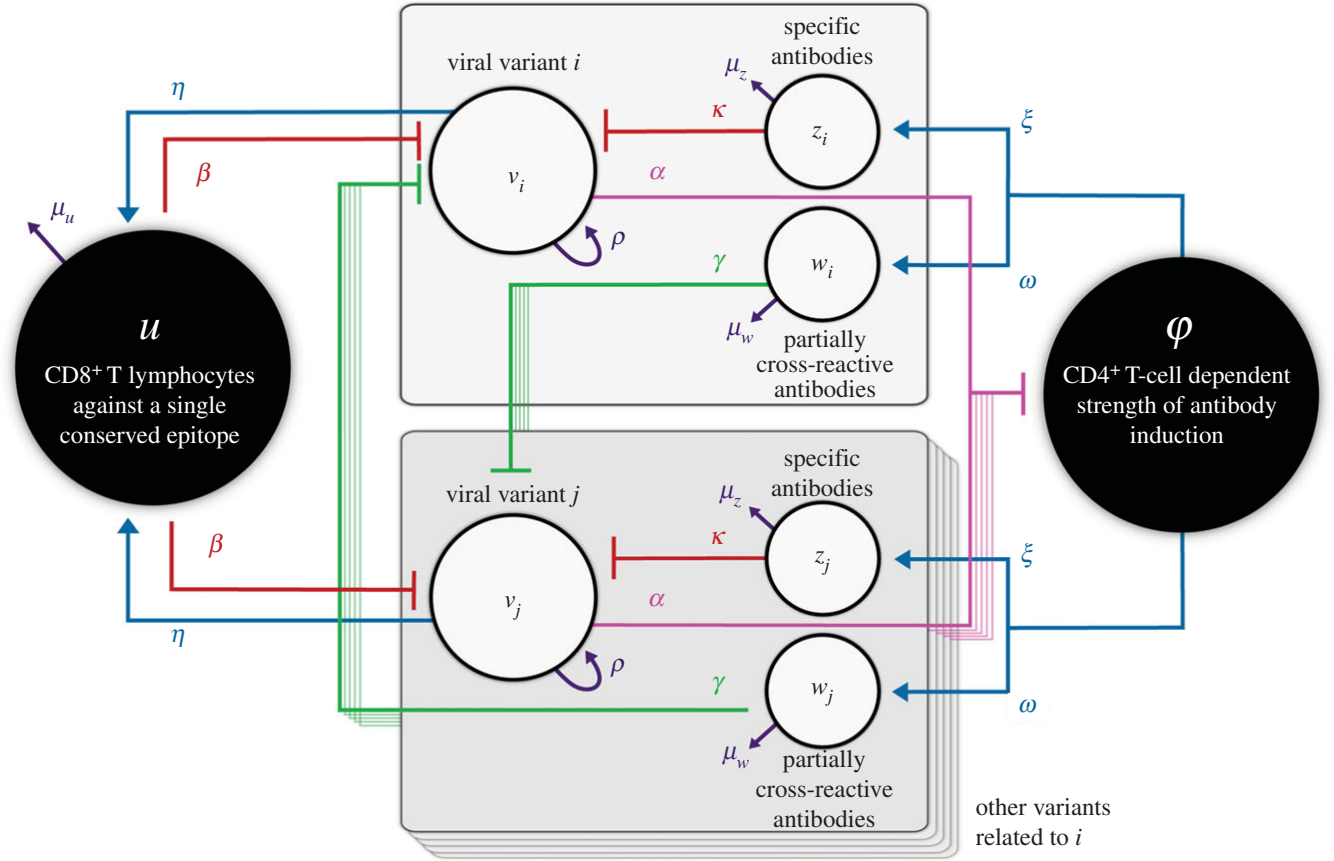
Model schematic. The population of viral variants of antigenic type *i* (*v_i_*) stimulates (shown by blue arrows) specific and partially cross-reactive antibodies as well as non-specific effector CD8^+^ T-cell responses. CD4^+^ T-cell help is essential for the induction of the antibody responses. *v_i_* can be attacked by all of these responses (shown by red bars) as well by partially cross-reactive antibodies (shown by green bars) raised against other variants *j* (stacked one behind the other) which share epitopes with *i*. CD4^+^ T-cells are attacked by all viral variants (as shown by pink bars): this is captured in the model by a reduction in CD4^+^ T-cell dependent strength of antibody induction, *φ*. All viral variants grow at a rate *ρ*; *μ_u_*, *μ_w_* and *μ_z_*, respectively, represent the death rates of effector CD8^+^ T cells, specific and partially cross-reactive antibodies. See §5 Material and methods for further details.

We visualize the virus as containing (i) CD8^+^ T-cell epitopes of limited variability that elicit cytotoxic responses [27] that decay rapidly in the absence of antigen [5,28], (ii) highly variable epitopes (specifically in the Env glycoprotein) that elicit both highly specific NAb responses maintained by long-lived plasma cells [29,30] and more broadly cross-reactive responses (CR-Ab) of shorter duration. Within our model, CD4^+^ T cells are necessary for the induction of the antibody responses but do not influence the induction of effector CD8^+^ T-cell responses (although they may have a role in the establishment of CD8^+^ T-cell memory). Finally, we assume that CD4^+^ cell counts decline at a rate proportional to viraemia. A schematic of the model structure is provided in figure 1 and the corresponding equations are shown in §5 Material and methods.

## 3. Results

### (a) Viral dynamics

**Figure 2.**
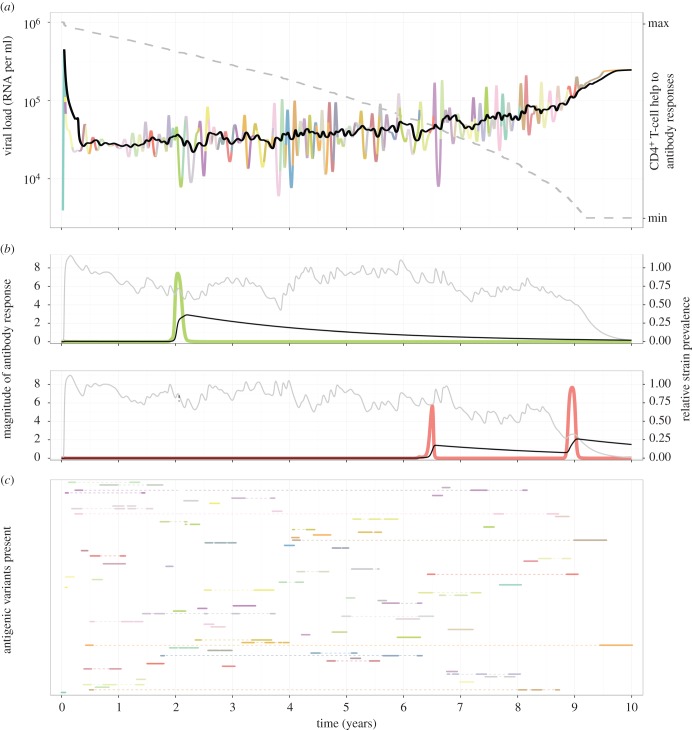
Viral dynamics. (*a*) Changes in viraemia (solid multicoloured line) and CD4^+^ T-cell help to antibody responses (dashed grey line) during the course of an infection, with the colour of the line illustrating which of the 81 possible antigenic variants is most prevalent at each time point. The black line shows a rolling average of viraemia over the three months preceding the timepoint or, when peak viraemia occurred less than three months earlier, the average over the period from peak viraemia to that timepoint. (*b***)** Changes with time in specific (black line) and cross-reactive (grey line) antibody responses (given in arbitrary units) against two particular antigenic variants whose relative prevalence is shown by green and red lines, respectively. (*c*) Each line tracks the prevalence of an antigenic variant with dots indicating a prevalence in excess of 10%. Re-emergence is indicated by a dashed line connecting periods of prevalence in excess of 10%. The two variants presented in (*b*) are shown by the dashed arrows. (Parameters: *ρ* = 8; 1/*μ_u_* = 10 days; 1/*μ_w_* = 100 days; 1/*μ_z_* = 1000 days; *β* = *γ* = *κ* = 1; *φ*(0) = 1; *η* = *ξ* = *ω* = 3.2×10^−5^; 1/*α* = 1.6×10^7^ days; antigenic variants are defined by combinations of four epitopes, each with three possible states, i.e. a {3,3,3,3} system.)

The observed dynamics of viraemia during the natural course of HIV-1 infection, with respect to three critical features, are readily generated under the minimal set of assumptions outlined above:

(i) The initial increase in viraemia triggers CD8^+^ T-cell responses as well as short-lived non-neutralizing partially cross-reactive antibody responses; highly specific NAb responses are induced at a slower rate as they have to undergo affinity maturation and therefore do not reach detectable levels until several months after infection [14,15]. Through a combination of these processes, a dynamic equilibrium is established in which viraemia fluctuates around a steady set-point, while CD4^+^ T-cell counts continue to decline (figure 2*a*).
(ii) When CD4^+^ T-cell counts drop to very low levels, antibody induction is compromised and a rapid transition occurs to a different dynamical state with a significantly higher viraemia corresponding to the clinical condition of AIDS (figure 2*a*). The difference in lifespan of effector CD8^+^ T cells as compared with NAb responses is the principal cause of the sharp increase in viraemia when the CD4^+^ T-cell count drops below a certain threshold (electronic supplementary material, figure S1*a*); this increase may be augmented by the loss of partially cross-reactive antibodies which also rely on CD4^+^ T-cell help for induction (electronic supplementary material, figure S1*b*,*c*). However, long-lived antibody responses tend to induce wider fluctuations around set-point (electronic supplementary material, figure S1*d*–*f*). At present, there is insufficient empirical data concerning variation in viral load during chronic infection to suggest which combination of CD8^+^ T cell and antibody lifespans most closely reproduces the dynamics of HIV-1, but empirical estimates (less than 50 days) for effector CD8^+^ T-cell responses [5,28,31–34] appear to lead to a relatively steady viral load under a wide ranges of values of both specific and cross-reactive antibody lifespans.
(iii) In line with empirical observations [14,15,29], the chronic phase of infection is characterized by the sequential dominance of antigenic variants of the Env glycoprotein (figure 2*b*,*c*), provided variant-specific NAbs are significantly longer lived than effector CD8^+^ T-cell responses (electronic supplementary material, figure S1*g*–*i*). Partially cross-reactive Ab responses (raised against related variants that have been recently prevalent) can significantly increase the tendency towards single strain dominance, as has been shown for other antigenically variable pathogens such as *Plasmodium falciparum* [35], although this may also lead to wider fluctuations in set-point viraemia (electronic supplementary material, figure S1*e*,*f*). Our model predicts that variants may re-emerge when cross-reactive and specific antibody responses directed against them fall below the required threshold (figure 2*b*,*c*): this is consistent with the observation that some viral variants that emerge late in chronic infection are susceptible to neutralization by contemporaneous NAbs, or to sera sampled much earlier in infection [36–39]. Within our framework, the loss of control of viraemia is characterized by the outgrowth of a small number of Env variants (electronic supplementary material, figure S2). If there is any variation in viral replicative capacity (VRC), then those with higher VRC are more likely to dominate; this could explain the trend towards an increase in VRC reported in the literature [40], but importantly is a consequence, rather than the cause, of the loss of viral control.

### (b) Dynamics of CD8^+^ T-cell escape

**Figure 3.**
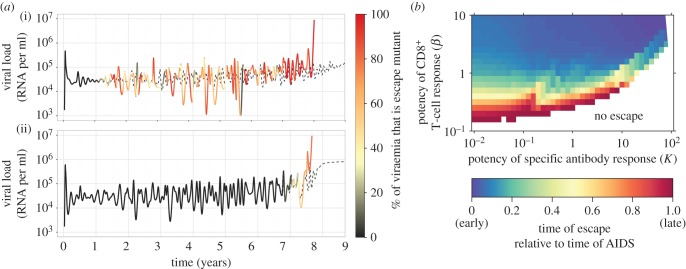
Escape from CD8^+^ T-cell responses. (*a*) The colour of the line indicates the fraction of the viral population composed of escape mutants (growth rate, *ρ_e_* = 7.6) or wild-type virus (*ρ* = 8.0), and changes from black (100% wild-type) to red (100% escape mutant). The dotted grey line shows the same time series where there is no escape possible from the CD8^+^ T-cell responses (*ρ_e_* = 0), and with otherwise exactly the same parameters (parameters are identical to figure 2, except *β* = 0.8 (*a*(i)) and *β* = 0.3 (*a*(ii)). (*b*) Ratio of time of escape to time of progression to AIDS and its dependence on the strength of antibody and CD8^+^ T-cell responses. Time of escape is defined as the earliest time that the escape mutant achieves more than 50% prevalence, and time of progression to AIDS is defined as the time that *φ* reaches 0 (parameters are identical to figure 2).

Escape from CD8^+^ T-cell responses can be included within our framework by allowing for mutations that abrogate recognition but at a cost to viral fitness (see §5 Material and methods). However, due to the complex interplay between antibody and CD8^+^ T-cell responses, these escape mutants may only spread through the viral population long after first being generated by mutation (figure 3) and fluctuate in frequency thereafter—as has been observed in both HIV [41] and SIV [42] infection. In essence, the weakening of antibody responses increases the relative selection pressure exerted by CD8^+^ T cells, steadily tipping the evolutionary balance in favour of CD8^+^ T-cell escape mutants and ultimately leading to their dominance. Strong antibody responses can therefore prevent escape from occurring for an extended period, even in the presence of highly efficacious CD8^+^ T-cell responses (figure 3*b*). It is also clear in this model that the emergence of escape mutants is neither necessary nor sufficient for the transition to AIDS, but does lead to faster disease progression by precipitating an increase in set-point viraemia [43,44] and a consequently more rapid loss of CD4^+^ T cells. Once escape has occurred, time to AIDS is principally dependent on the potency of the antibody response (which explains why it is so similar between the examples of early and late escape shown in figure 3*a*), but may also be significantly affected by the relative fitness of the escape mutant (electronic supplementary material, figure S4).

## 4. Discussion

A number of mathematical models have been proposed for the pathogenesis of HIV-1, variously linking the loss of control of viraemia to the accumulation of antigenic diversity [45], gradual immune escape [46], enhanced viral growth rates [47], accumulation of deleterious mutations in thymocytes due to over-exertion of the immune system [48], progressive dendritic cell dysfunction [49] or a consequence of a homeostatic mechanism that acts to balance CD4^+^ and CD8^+^ T-cell numbers [50]. Here, we propose a simple alternative framework that explains many important aspects of HIV-1 pathogenesis by combining the effects of long-lived variant-specific antibodies alongside short-lived effector CD8^+^ T-cell responses. Importantly, in our model, it is the loss of antibody induction that triggers a shift in the dynamical state of the system causing a nonlinear increase in viraemia during transition to AIDS.

It is important to note that the model presented in this paper belongs within a well-established tradition of conceptual mathematical modelling within population biology and epidemiology (e.g. [45]), where the principal aim is to elucidate the key interactions that underlie population dynamics rather than to make specific quantitative predictions. Accordingly, the practices of parametrization we have followed (see §5 Material and methods) do not directly correspond to those employed within predictive models, because our aims are fundamentally different. The key question we are asking is whether differences in lifespan of cytotoxic responses against less variable CD8^+^ T-cell epitopes and of antibody responses against more variable B cell epitopes can combine in such a manner as to reproduce the dynamics of HIV-1 infection (see the electronic supplementary material); other parameters have been set to produce realistic levels of set-point viraemia. It is crucial to acknowledge that the qualitative conclusions would remain unaltered under a different choice of parameters for viral growth rate and induction and killing rates of the respective immune responses: the validity of a conceptual model is not reliant on selecting parameters to provide an exact match with empirical data. We have provided a mathematical analysis (see the electronic supplementary material) to underline this point.

An important implication of our model results is that an increase in potency or strength of induction of the antibody response has much more profound consequences for set-point viraemia, and hence disease progression, than a similar increase in relative magnitude or efficacy of CD8^+^ T-cell responses (electronic supplementary material, figures S3 and S5). A subset of HIV-1 infected individuals, known as long-term non-progressors, remain asymptomatic for many years with high CD4^+^ counts (more than 500 cells μl^−1^) and low plasma HIV-RNA levels (less than 10 000 copies ml^−1^) [51]; within our model, this can arise solely as a consequence of greater overall effectiveness of CD8^+^ T-cell responses and difficulty of escape. However, a more dramatic decrease in viraemia, as observed among elite controllers (ECs) of HIV-1 infection (less than 50 copies ml^−1^), is difficult to attribute to stronger CD8^+^ T-cell responses alone. Indeed, many ECs do not possess any of the canonically beneficial HLA class I alleles [52] and demonstrate extensive escape from CD8^+^ T-cell responses [53,54]. Differences in set-point can be readily achieved within our framework by lowering VRC; however, ECs are often found to be infected with replication competent viruses [55,56]. These observations are easily reconciled within our model, and we predict that in ECs stronger NAb responses alone can be enough to substantially reduce viraemia.

The role of antibodies in the control of HIV has been questioned by the observation that NAb titres do not appear to decline prior to the loss of control of the set-point viraemia (e.g. [57]). Our results illustrate that NAbs cannot be discounted as a mediator of potent viral control on this basis since, as shown in figure 2*b*, if NAb responses are long-lived, their titres may be expected to decline only slowly after control of viral replication has been lost. It is also worth noting that individuals with more potent NAb responses will also have lower levels of circulating antibody (as they control their viraemia more successfully) and thus may not always evince a higher titre than someone who has less potent and consequently higher levels of circulating antibody. Certainly, ECs have been shown to display equivalent NAb titres to normal progressors [58].

The rapid turnover and limited coexistence of viral lineages shown by phylogenetic analyses of early-phase HIV-1 diversity [59] are compatible with the strong sequential dominance of variants exhibited by this model (figure 2*a*). It is important to note that the antigenic types whose dynamics are described here cannot be easily equated with current sequence data, as the same antigenic phenotype can correspond to multiple sequences, which need not be adjacent in sequence space. An extreme example of this is the appearance and disappearance of N-linked glycosylation sites, whereby a single-nucleotide polymorphism can have strong effects on antigenic phenotype by masking epitopes [36]. Subsequent reversion of this single mutation would abrogate glycosylation, resulting in the re-emergence of the original antigenic phenotype, but divergence accrued elsewhere in the genome would mean that this later isolate would inevitably occupy a very different phylogenetic location. The outgrowth of only one or a few Env variants upon transition to AIDS is also consistent with current data on HIV evolution: the fixation rate of non-synonymous mutations remains high during chronic infection as a result of the continual molecular adaptation arising from Env variant turnover [56] but declines significantly upon the transition to AIDS [60].

Within our framework, partially cross-reactive antibodies have a significant impact on viral dynamics. It is important to distinguish these from the slowly developing broadly neutralizing antibodies that are currently being considered as vaccine targets [61]. Rather, the former represent a rapidly developing non-neutralizing response with Fc-related activities, such as antibody-dependent cellular cytotoxicity or antibody-mediated cellular viral inhibition (ADCVI), and are likely to be directed at epitopes of intermediate variability. Recent studies [62] suggest that these responses peak early but then decline; our model suggests that they nonetheless continue to play a crucial role in preventing the diversification of the viral population. This is supported by studies in rhesus monkeys showing that viruses isolated in the chronic phase of SIV infection, and against which there is no detectable contemporaneous, autologous neutralizing response, remain susceptible to ADCVI responses in plasma from much earlier in the infection [63]. Vaccine strategies based around the boosting and maintenance of these partially cross-protective responses could therefore be strongly beneficial in preventing disease progression; we note that the modest protection observed in the RV144 vaccine trial was correlated with (non-neutralizing) antibody binding titres to the V1V2 domain [64].

By linking the loss of control of viraemia to the failure of antibody induction, we solve several problems that arise when attempting to connect progression to disease with loss of CD8^+^ T cells. However, our model does not discount the role of CD8^+^ T cells in *delaying* progression. The well-established link between HLA Class I type and disease progression [9–13] is explained within our framework as a direct consequence of the differences among HLA types in the strength of their CD8^+^ T-cell responses and the fitness of associated escape mutants. More effective CD8^+^ T-cell responses have the effect of lowering set-point and thereby delaying the decline in CD4^+^ T cells that are essential to the maintenance of the antibody response. Although suggested by a number of studies (e.g. [65]), the precise relationship between viraemia and rate of decline in CD4^+^ is not fully understood [66,67]. While such a link is not strictly essential in our model, this additional assumption provides the correlation between viral set-point and time to AIDS that is widely observed among HIV-1 infections.

Our model also highlights the significant impact of increasing CD8^+^ T-cell effector lifespan on time to AIDS (electronic supplementary material, figure S6). Indeed, extension of CD8^+^ T-cell lifespan may also underlie the protective role of inhibitory killer cell immunoglobulin-like receptors, such as KIR3DL1 [68], which have been reported to reduce activation-induced CD8^+^ T-cell death in a number of chronic viral infections [69]. Furthermore, by limiting damage to gut-associated lymphoid tissue in early infection [1,70], a stronger CD8^+^ T-cell response could also have an effect on the quality of antibody responses, thereby indirectly contributing to low viraemia. The complex interplay between antibody and CD8^+^ T-cell responses can lead to a wide distribution of times before an escape mutant eventually outcompetes the wild-type (figure 3), and strong antibody responses can prevent escape from occurring for an extended period, even in the presence of highly efficacious CD8^+^ T-cell responses. Caution must therefore be exercised in interpreting late escape as an indication of poor CD8^+^ T-cell control. We urge that most such empirical observations relating to the within-host dynamics of HIV-1 should be understood in the context of a framework that integrates B-cell and T-cell responses and represents them in terms of the variability of their targets and differences in the lifespans of the effectors.

## 5. Material and methods

Our model may be described by the following set of ordinary differential equations:5.1
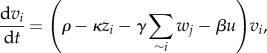
5.2
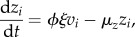
5.3
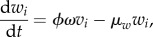
5.4
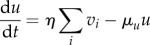
5.5
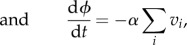
where *v_i_* is the viral load associated with variant *i*; *z_i_* and *w_i_*, respectively, denote the levels of specific and partially cross-reactive antibodies elicited by this variant; *u* represents the level of CD8^+^ T lymphocytes against a single conserved epitope; the decay rates of these responses are designated, respectively, by *μ_z_*, *μ_w_*, *μ_u_*; *ξ*, *ω* and *η* are associated baseline induction rates; *κ*, *γ* and *β* are the associated rates of killing; *ρ* is the viral growth rate; *φ* measures the remaining ability to make new antibody responses; *j* designates strains that share antibody epitopes with *i*. We can represent these shared epitopes using a multilocus structure {*m*_1_, *m*_2_,…*m_n_*}, where *m_x_* describes the number of alleles at locus *x*, and *n* represents the total number of loci [71]; we assume that the particular combination of shared epitopes represented by *i* uniquely determines the specificity of the long-lived NAb response against this variant. The dynamics of CD4^+^ T-cell count are included by allowing the strength of specific antibody induction, *φ*, to decline in proportion to total viral load at a rate *α*.

We consider the impact of escape from CD8^+^ T-cell responses in the model by supposing that, for each *i*, there is a mutant strain, *v_e_*_,*i*,_ with reduced viral growth rate, *ρ_e_*, which cannot be recognized or targeted by CD8^+^ T-cell responses, introducing the additional equation:5.6
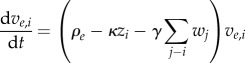


The other equations may be altered accordingly to give5.7
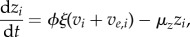
5.8
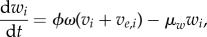
5.9
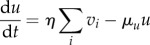
5.10
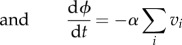


## Supplementary Material

Supplementary Figures and Mathematical analysis
