# Supplementary material for: Effects of neutralizing antibodies on escape from CD8+ T-cell responses in HIV-1 infection
Source: Philos Trans R Soc Lond B Biol Sci. 2015 Aug 19;370(1675):20140290. doi: 10.1098/rstb.2014.0290 (PMC4528488; doi:10.1098/rstb.2014.0290)
Supplement: Supplementary Figures and Mathematical analysis [file rstb20140290supp1.pdf]

Wikramaratna, PS. *et al.* 2015. Effects of neutralising antibodies on escape from CD8+ T cell responses in HIV-1 infection. *Phil. Trans. R. Soc. B* **370** doi: 10.1098/rstb.2014.0290

Supplementary Material

1. Dependence of viral dynamics on duration of immune responses

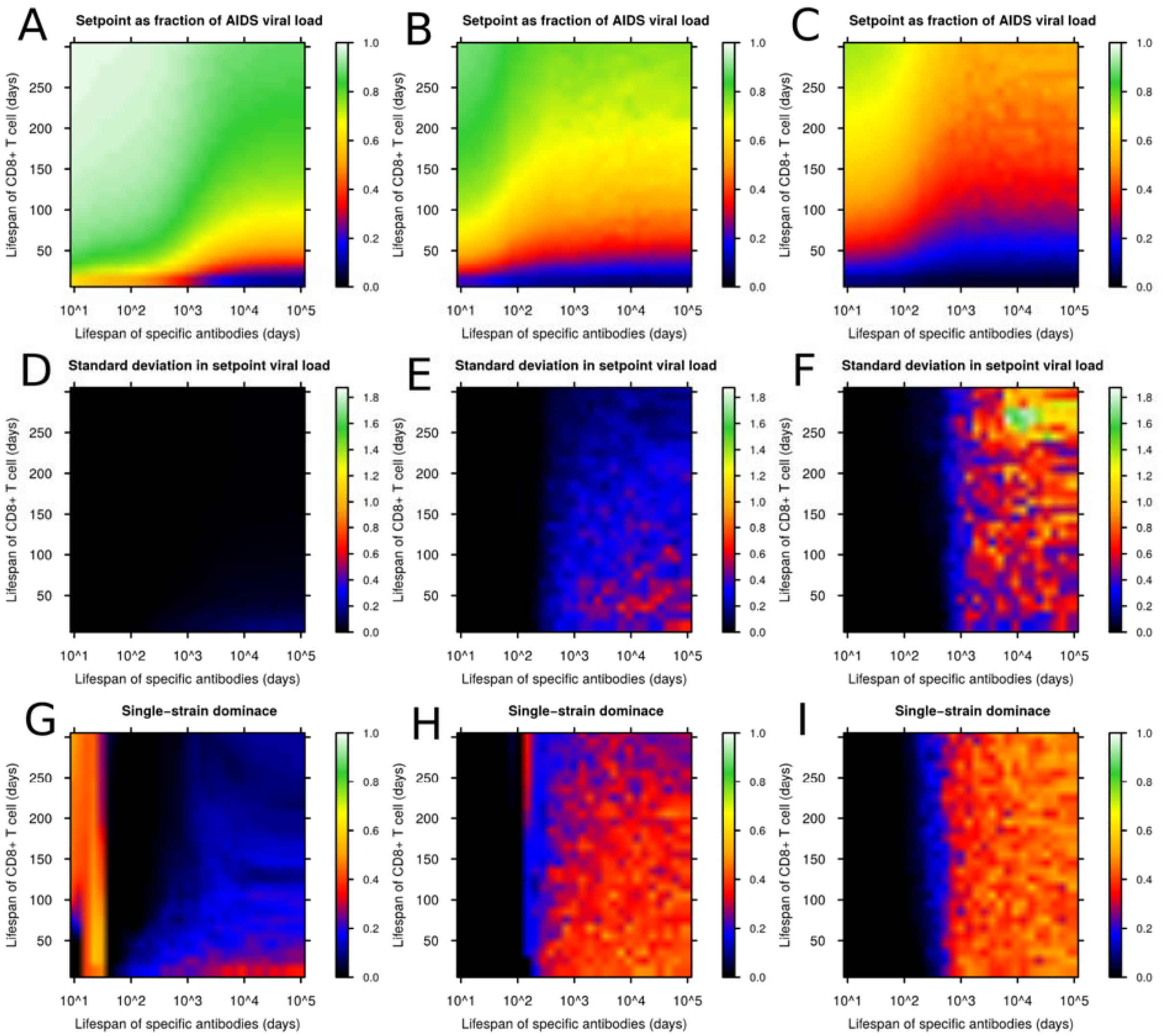

Figure S1: Realistic HIV-1 dynamics can be generated under a combination of short-lived CD8+ T cell and long-lived specific antibodies, with short-lived cross-reactive antibodies promoting sequential dominance of variants. Shown here are the effects of CD8+ T cell and specific antibody lifespan on (i) ratio of set-point viral load to final (AIDS) viral load (a-c), (ii) standard deviation in set-point viral load (d-f) and (iii) single strain dominance (g-i) with lifespan of partially cross-reactive response increasing from 10 to 100 to 300 days from left to right column. Other parameters and initial conditions identical to Figure 2 in main text. Single strain dominance can be quantified by the measure ε by comparing the relative prevalence of the two most common antigenic variants within single epidemics, and then averaging across extended periods of time (see Recker *et al*, 2007 *Proc Natl Acad Sci USA* 104: 7711-7716).

2. Antigenic dynamics of late infection

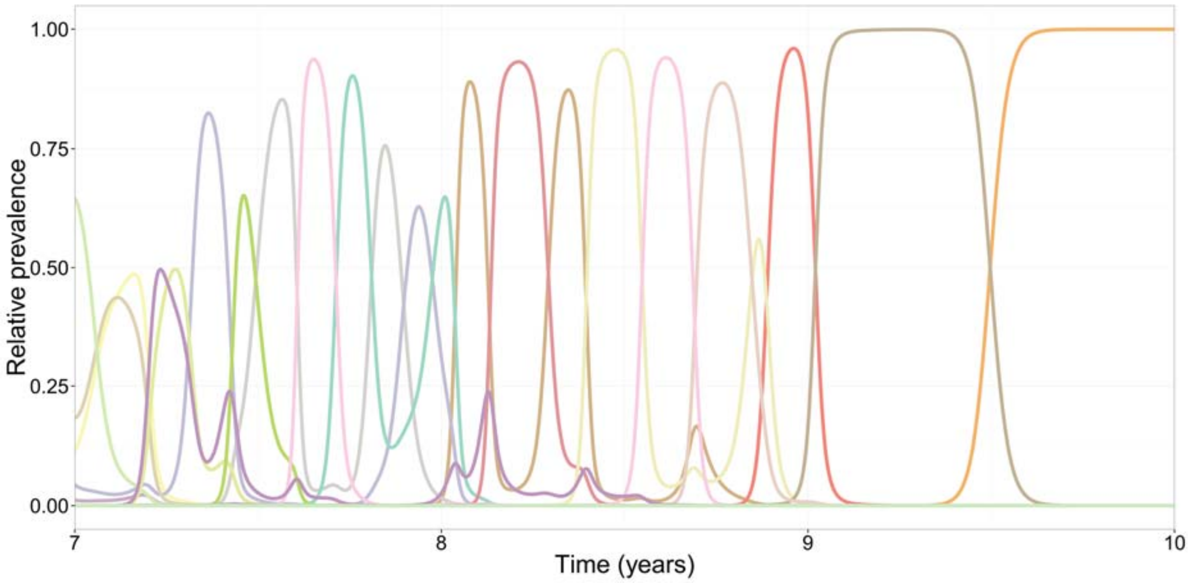

Figure S2: Prevalence of different variants in late infection Each coloured line represents the prevalence (plotted as a fraction of total viraemia) of a different antigenic variant. Parameters and initial conditions identical to Figure 2 (main text).

3. Sensitivity of setpoint viraemia to potency of specific antibody and CD8+ T cell responses

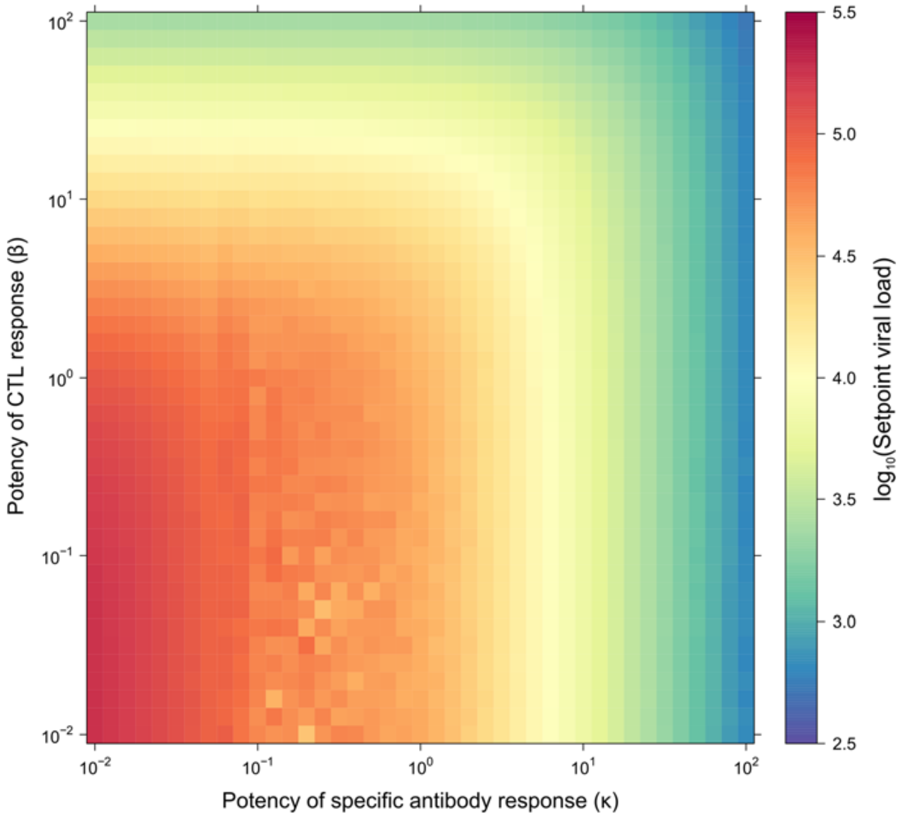

Figure S3: Setpoint viraemia is more sensitive to potency of Nab responses than it is to the potency of CTL responses. Setpoint viraemia was calculated by running the model in the absence of immune decay for 8 years, and taking the average viraemia for years 6-8 (Other parameters: $\rho = 8$; $1/\mu_u = 10$ days; $1/\mu_w = 100$ days; $1/\mu_z = 1000$ days; $\gamma = 1$; $\varphi(0) = 1$; $\eta = \xi = \omega = 3.2*10^{-5}$; $\alpha = 0$ days^{-1}; n=4, m=3).

4. Effect of escape on Time to AIDS

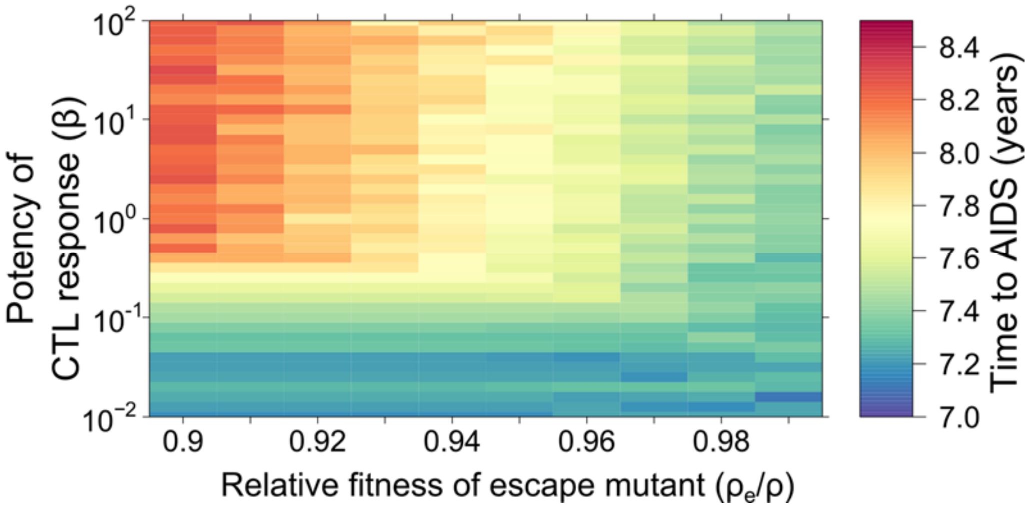

Figure S4: Time to AIDS is affected both by the potency of CD8+ T cell responses and the relative fitness of the escape mutant. Time of progression to AIDS is defined as the first time that φ reaches 0. (Constant parameters: $\rho = 8$; $1/\mu_u = 10$ days; $1/\mu_w = 100$ days; $1/\mu_z = 1000$ days; $\gamma = \kappa = 1$; $\varphi(0) = 1$; $\eta = \xi = \omega = 3.2*10^{-5}$; $\alpha = 0$ days^{-1}).

5. Mathematical analysis

We are able to explain the basic behaviour of the model by analysing the system in the absence of variability in the virus population. Under these circumstances, the equations can be reduced to:

$$\frac{dv}{dt} = (\rho - \kappa z - \beta u)v \quad \text{(S1)}$$

$$\frac{dz}{dt} = \phi v - \mu_z z \quad \text{(S2)}$$

$$\frac{du}{dt} = \eta v - \mu_u u \quad \text{(S3)}$$

This reduced system admits two steady states within (v, z, u) space: C_0 = (0, 0, 0) and C* = (v*, z*, u*) given by:

$$v^* = \frac{\rho \mu_u \mu_z}{\phi \kappa \mu_u + \eta \beta \mu_z} \qquad z^* = \frac{\rho \phi \mu_u}{\phi \kappa \mu_u + \eta \beta \mu_z} \qquad u^* = \frac{\rho \eta \mu_z}{\phi \kappa \mu_u + \eta \beta \mu_z}$$

The eigenvalues $(\lambda_1, \lambda_2, \lambda_3)$ of the Jacobian matrix are the roots of the polynomial:

$$\left(\lambda - (\rho - \kappa z - \beta u)\right)(\lambda + \mu_z)(\lambda + \mu_u) + \phi \kappa v(\lambda + \mu_z) + \eta \beta v(\lambda + \mu_u) = 0$$

Thus for C_0 we find: $\lambda_1 = \rho; \lambda_2 = -\mu_z; \lambda_3 = -\mu_u$ and hence C_0 is a saddle point.

Substituting for v*, z* and u* in C* reveals that the eigenvalues satisfy:

$$\lambda^3 + (\mu_z + \mu_u)\lambda^2 + (\mu_z \mu_u + \phi \kappa v^* + \eta \beta v^*)\lambda + \phi \kappa \mu_z v^* + \eta \beta \mu_u v^* = 0$$

Each coefficient in this expression is positive, thus each eigenvalue negative and C* stable.

We can further interpret v* (i.e. the steady state viral load in the absence of immune decay) as the set-point viral load.

One of the chief assumptions of this model is that the NAb response has much greater longevity than the CD8+ response; v* is thus much more sensitive to variation in antibody

induction and potency (ϕ, κ) than it is to CD8+ induction and potency (η, β) as shown in Figure S5.

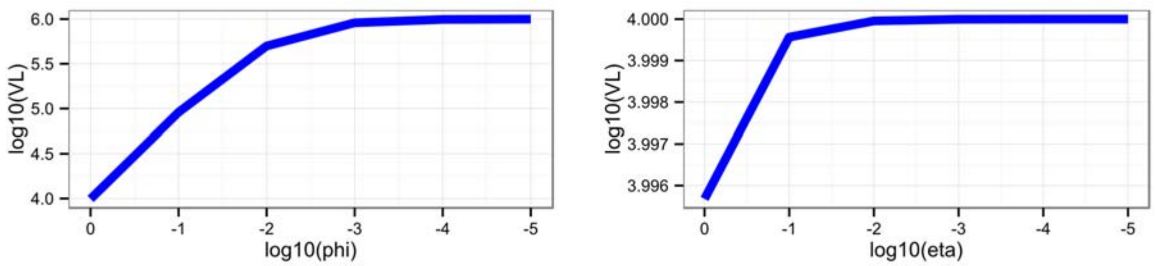

Figure S5. Effect on v* of decreasing strength of (A) antibody induction, (B) CD8+ T cell induction ($\rho = 10^6$; $1/\mu_u = 1$ day; $1/\mu_z = 1000$ days; $\kappa = 1$; $\eta = 1$; $\beta = 1$; and $\eta = 1$ [left], $\phi = 1$ [right])

We can consider the effect of CD4+ T cell decay in this model by introducing an equation:

$$\frac{d\phi}{dt} = -\alpha \qquad (\phi > 0) \qquad \text{(S4)}$$

From Figure S1a, it is evident that while $\phi \gg 0$, $v^*(\phi)$ is roughly constant, but experiences a sharp decline as $\phi \to 0$; thus these few assumptions alone can generate the 3-phase pattern of viraemia associated with HIV infection (Figure 2a).

Increasing the lifespan of CD8+ T cell effector response will also reduce setpoint viral load (and hence increase the time it takes to progress to AIDS in the model).

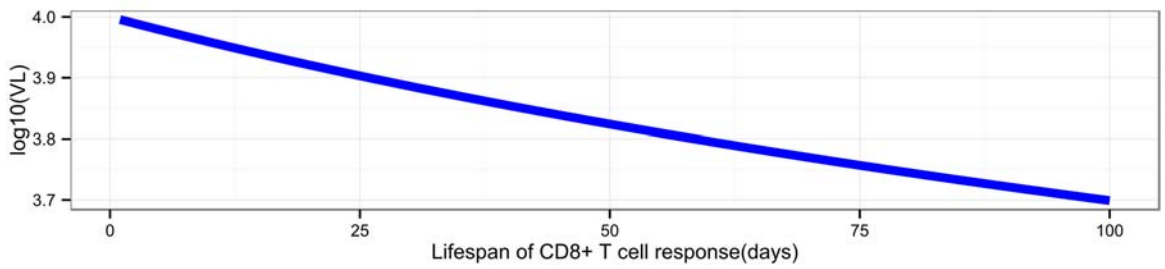

Figure S6. Change in v*, as a function of the lifespan of the CD8+ T cell response ($1/\mu_u$). Other parameters: $\rho = 10^6$; $1/\mu_z = 1000$ days; $\kappa = 1$; $\eta = 1$; $\beta = 1$; $\eta = 1$; $\phi = 1$.

Assuming that the rate of decay of φ is proportional to total viral load (equation 5) does not alter the basic behaviour of the model, but allows us to recover the known relationship between set-point viraemia and time to AIDS. Linking φ to viral load in this manner also causes it to decline significantly in the early stages of infection (see Fig 2a in main text) and

thus captures the widespread depletion of mucosal CD4+ T cells and irreversible destruction of the GALT occurring during this period.

Notably, our model is able to capture such dynamics without including any saturating terms or limitations in resource availability such as the availability of CD4+ T cells for infection. Rather, the achievement of set-point and ultimate progression to AIDS both follow from a balance of immune responses. The inclusion of such limiting terms would be unlikely to alter the qualitative dynamics of the model, but may act to quantitatively affect the magnitude of peak viraemia in acute infection.
